# Matched and mismatched unrelated donor compared to autologous stem cell transplantation for acute myeloid leukemia in first complete remission: a retrospective, propensity score-weighted analysis from the ALWP of the EBMT

**DOI:** 10.1186/s13045-016-0314-x

**Published:** 2016-09-02

**Authors:** Francesco Saraceni, Myriam Labopin, Norbert-Claude Gorin, Didier Blaise, Reza Tabrizi, Liisa Volin, Jan Cornelissen, Jean-Yves Cahn, Patrice Chevallier, Charles Craddock, Depei Wu, Anne Huynh, William Arcese, Mohamad Mohty, Arnon Nagler, Acute Leukemia Working Party (ALWP) of the European society for Blood and Marrow Transplantation (EBMT)

**Affiliations:** 1 Hematology and Bone Marrow Transplantation, Polytechnic University of Marche—Ospedali Riuniti Ancona, Via Conca 71, 60126 Ancona, Italy; 2 ALWP-EBMT and Department of Hematology and Cell Therapy, Saint Antoine Hospital, Paris, France; 3 Programme de Transplantation et Therapie Cellulaire—Institut Paoli Calmettes, Marseille, France; 4 CHU Bordeaux, Hôpital Haut-Leveque, Pessac, France; 5 HUH, Comprehensive Cancer Center, Stem Cell Transplantation Unit, Helsinki, Finland; 6 Erasmus MC-Daniel den Hoed Cancer Centre, Rotterdam, The Netherlands; 7 Clinique Universitaire d’Hématologie CHU Grenoble, Grenoble, France; 8 Department D’Hématologie, CHU Nantes, Nantes, France; 9 Centre for Clinical Haematology, Queen Elizabeth Hospital, Birmingham, UK; 10 Department of Hematology, First Affiliated Hospital of Soochow University, Suzhou, China; 11 CHU Department Hématologie, Hôpital de Purpan, Toulouse, France; 12 Rome Transplant Network, Stem Cell Transplant Unit, Tor Vergata University of Rome, Rome, Italy; 13 Chaim Sheba Medical Center, Tel-Hashomer, Israel; 14 ALWP-EBMT Office, Saint Antoine Hospital, Paris, France

**Keywords:** Acute myeloid leukemia (AML), Allogeneic transplantation, Matched (10/10) and mismatched (9/10) unrelated donor transplantation, Autologous transplantation, Post-remission therapy

## Abstract

**Background:**

Optimal post-remission strategy for patients with acute myeloid leukemia (AML) is matter of intense debate. Recent reports have shown stronger anti-leukemic activity but similar survival for allogeneic stem cell transplantation (allo-HSCT) from matched sibling donor compared to autologous transplantation (auto-HSCT); however, there is scarcity of literature confronting auto-HSCT with allo-HSCT from unrelated donor (UD-HSCT), especially mismatched UD-HSCT.

**Methods:**

We retrospectively compared outcome of allogeneic transplantation from matched (10/10 UD-HSCT) or mismatched at a single HLA-locus unrelated donor (9/10 UD-HSCT) to autologous transplantation in patients with AML in first complete remission (CR1). A total of 2879 patients were included; 1202 patients received auto-HSCT, 1302 10/10 UD-HSCT, and 375 9/10 UD-HSCT. A propensity score-weighted analysis was conducted to control for disease risk imbalances between the groups.

**Results:**

Matched 10/10 UD-HSCT was associated with the best leukemia-free survival (10/10 UD-HSCT vs auto-HSCT: HR 0.7, *p* = 0.0016). Leukemia-free survival was not statistically different between auto-HSCT and 9/10 UD-HSCT (9/10 UD-HSCT vs auto-HSCT: HR 0.8, *p* = 0.2). Overall survival was similar across the groups (10/10 UD-HSCT vs auto-HSCT: HR 0.98, *p* = 0.84; 9/10 UD-HSCT vs auto-HSCT: HR 1.1, *p* = 0.49). Notably, in intermediate-risk patients, OS was significantly worse for 9/10 UD-HSCT (9/10 UD-HSCT vs auto-HSCT: HR 1.6, *p* = 0.049), while it did not differ between auto-HSCT and 10/10 UD-HSCT (HR 0.95, *p* = 0.88). In favorable risk patients, auto-HSCT resulted in 3-year LFS and OS rates of 59 and 78 %, respectively.

**Conclusions:**

Our findings suggest that in AML patients in CR1 lacking an HLA-matched sibling donor, 10/10 UD-HSCT significantly improves LFS, but this advantage does not translate in better OS compared to auto-HSCT. In intermediate-risk patients lacking a fully HLA-matched donor, auto-HSCT should be considered as a valid option, as better survival appears to be provided by auto-HSCT compared to mismatched UD-HSCT. Finally, auto-HSCT provided an encouraging outcome in patients with favorable risk AML.

**Electronic supplementary material:**

The online version of this article (doi:10.1186/s13045-016-0314-x) contains supplementary material, which is available to authorized users.

## Background

Optimal post-remission strategy for patients with acute myeloid leukemia (AML) is a matter of debate. Allogeneic stem cell transplantation (allo-HSCT) is the most effective treatment to prevent leukemia relapse, and for patients lacking a matched sibling donor (MSD), transplantation from a 10/10 matched unrelated donor (MUD) is the preferred alternative [1]. The indication for allo-HSCT from 9/10 unrelated donor is more controversial, and outcome according to patient and disease characteristics has not been fully established yet [2].

Autologous stem cell transplantation (auto-HSCT) is an alternative approach, which was initially designed to consolidate remission in AML patients lacking a sibling donor or unfit for allo-HSCT [3]; since then, auto-HSCT passed through alternate fortunes, and its use progressively declined following evolution of allo-HSCT protocols [1, 4, 5]. Nevertheless, auto-HSCT holds several advantages including low non-relapse mortality rates, absence of graft-vs-host disease (GVHD) risk, lower incidence of late effects, and better quality of life for survivors compared to allo-HSCT; concerns include high relapse rate, due to the absence of *graft-vs-leukemia* (GVL) effect and the theoretic possibility of graft contamination by leukemic cells [6].

Recent reports [7–9] comparing allo-HSCT and auto-HSCT evidenced similar survival and concluded that auto-HSCT should still be considered as a valid alternative to allo-HSCT and taken into account within AML post-remission strategies. Therefore, since transplants from unrelated donors (UD) are currently the preferred option worldwide, and given the lack of a study confronting auto-HSCT with mismatched UD-HSCT, we took the advantage of the European society for blood and marrow transplantation (EBMT) data set and retrospectively compared outcome of matched (10/10 UD-HSCT) or mismatched at a single HLA-locus unrelated donor transplantation (9/10 UD-HSCT) with auto-HSCT in patients with AML in first complete remission (CR1).

## Methods

### Study design and data collection

This is a retrospective multicenter study. Data were provided, and the study design was approved by the acute leukemia working party (ALWP) of the EBMT group registry, in accordance with the EBMT guidelines for retrospective studies. EBMT is a voluntary working group of more than 500 transplant centers which are required to report all consecutive stem cell transplantations and follow up once a year (Additional file 1). Audits are routinely performed to determine the accuracy of the data. We included in the analysis patients affected by AML older than 18 at diagnosis, who received either auto-HSCT, 10/10 UD-HSCT, or 9/10 UD-HSCT in CR1 as first transplant between January 2005 and December 2013. Patients having secondary AML were excluded. Only patients with available cytogenetic data and allelic HLA typing for A, B, C, DRB1, and DQB1 (for UD-HSCT) were included. Good risk was defined as *t*(8,21), inv(16)/*t*(16;16), or normal karyotype in the presence of NPM1 mutation without fms-like tyrosine kinase-internal tandem duplication (FLT3-ITD). Poor risk was defined as −7, abnl (17p) −5/5q−, inv(3q)/*t*(3;3), *t*(6;9), *t*(v;11)(v;q23), MLL rearranged except of *t*(9;11)(p22;q23), complex karyotype, or normal karyotype in the presence of FLT3-ITD. Intermediate risk was defined as *t*(9;11)(p22;q23), normal karyotype without NPM1 or FLT3-ITD, or the absence of abnormalities categorized as good or poor risk [10]. One hundred and twenty patients receiving auto-HSCT, 217 10/10 UD-HSCT, and 60 9/10 UD-HSCT had normal karyotype and wild-type FLT3 (*wt*FLT3) and were analyzed separately as “intermediate *wt*FLT3” group. Nine hundred and forty-two patients (504 auto-HSCT, 333 10/10 UD-HSCT, and 105 9/10 UD-HSCT) had normal karyotype and unknown molecular markers and were therefore assigned to the intermediate-risk group. Patients from 283 transplant centers were included; 83 centers reported both auto-HSCT and UD-HSCT. One thousand six hundred thirteen patients were transplanted in centers having reported both auto-HSCT (*n* = 890) and UD-HSCT (*n* = 723), while 1266 patients in centers having reported only auto-HSCT (*n* = 787) or UD-HSCT.

### Endpoint definitions and statistical analysis

Endpoints were relapse incidence (RI), non-relapse mortality (NRM), leukemia-free survival (LFS), and overall survival (OS). Cumulative incidences of relapse and NRM were calculated from the date of transplant to the date of relapse or death, respectively, with the other events being the competing risk. LFS was defined as the interval from transplant to either relapse or death. OS was defined as the time between the date of transplant and the date of death.

The main patient characteristics were compared using Mann-Whitney test for quantitative variables, chi-square test, or Fisher’s exact test for categorical variables. We used propensity score (PS) weighting to control for pre-treatment imbalances on observed variables. The following factors were included in the PS model: age, year of transplant, interval diagnosis transplant, number of induction courses to reach CR1 (1 vs more than 1), and cytogenetic risk. PS estimation was performed using generalized boosted models [11]. As the research question focused on the effectiveness of 10/10 UD-HSCT or 9/10 UD-HSCT if it were to replace auto-HSCT for patients having the same characteristics of those actually receiving auto-HSCT, we weighted the 10/10 UD-HSCT and 9/10 UD-HSCT groups to match the auto-HSCT group, by estimating the average treatment effect among the treated (ATT), auto-HSCT being the treated group. The ATT weights equal one for auto-HSCT, and it equals the ratio of the PS to one minus the PS in the two UD-HSCT groups. In summary, each patient that underwent UD-HSCT received a weight inversely proportional to his probability of receiving an autograft. Therefore, patients receiving UD-HSCT that showed significantly different characteristics compared to average autografted patients had a low contribution in the comparisons. We checked the balance between the groups looking to ATT-weighted means. Then, we used pairwise ATTs to fit weighted Kaplan-Meier and Cox models separately for auto-HSCT vs 10/10 UD-HSCT and auto-HSCT vs 9/10 UD-HSCT, adjusting for stem cell source (bone marrow or peripheral blood stem cells) and conditioning regimen (total body irradiation-based or not). The same procedure was repeated for each cytogenetic-risk group. Finally, we looked to the subgroup of patients with intermediate cytogenetics and wild-type FLT3, adding the time interval from CR1 to transplant to the PS model. All the results were checked by performing a subanalysis excluding the fourth percentile for the interval from diagnosis to transplant, obtaining consistent results. All tests were two-sided. The type I error rate was fixed at 0.05 for determination of factors associated with time to event. Analyses were performed using the R statistical software version 3.2.3; PS analysis was performed using the mnps function of the Twang package and weighted analyses using the survey package [12].

## Results

### Patient characteristics

The total number of patients who received either auto-HSCT or UD-HSCT for AML in CR1 between 2005 and 2013 and reported to the EBMT was 8943 (3161 auto-HSCT and 5782 UD-HSCT). One thousand nine hundred and fifty-eight patients were excluded from the analysis due to incomplete data about HLA typing. Ninety-six patients were excluded as received UD-HSCT which was 8/10 HLA-matched or inferior, leading to a total number of 6889 patients available for analysis of outcome (3161 auto-HSCT, 2921 10/10 UD-HSCT, and 807 9/10 UD-HSCT). Finally, 4010 patients were subsequently excluded due to incomplete data about cytogenetics, leading to a final number of 2879 patients included in the propensity score model. Among them, 1202 received auto-HSCT, 1302 10/10 UD-HSCT, and 375 9/10 UD-HSCT, respectively. Median follow-up was 45, 36, and 34 months for auto-HSCT, 10/10 UD-HSCT, and 9/10 UD-HSCT, respectively. Median age at transplant was higher for 10/10 UD-HSCT (51 years) compared to 9/10 UD-HSCT and auto-HSCT (49 years for 9/10 UD-HSCT and auto-HSCT, *p* = 0.004). Interval from diagnosis to transplant was longer for UD-HSCT (174 and 177 days for 10/10 UD-HSCT and 9/10 UD-HSCT, respectively) compared to auto-HSCT (158 days, *p* < 10^−4^). Patients who received UD-HSCT showed more frequently poor-risk cytogenetics (16, 47, and 49 % for auto-HSCT, 10/10 UD-HSCT, and 9/10 UD-HSCT, respectively, *p* < 10^−4^) and were more likely to have received a total body irradiation (TBI)-based conditioning (*p* < 10^−4^). Median year of transplant was more recent for UD-HSCT (2010) compared to auto-HSCT (2008, *p* < 10^−4^). Stem cell source was peripheral blood stem cells for 96 % of auto-HSCT, 80 % of 10/10 UD-HSCT, and 85 % of 9/10 UD-HSCT patients (*p* < 10^−4^). Among the UD-HSCT cohort, 813 patients received a myeloablative (MAC) conditioning (619 in the 10/10 UD-HSCT and 194 in the 9/10 UD-HSCT group, respectively), while 857 received a reduced-intensity (RIC) conditioning regimen (677 in the 10/10 UD-HSCT and 180 in the 9/10 UD-HSCT group). The characteristics of the patients are summarized in Table 1.

**Table 1:** Patient, disease, and transplant characteristics

	Type of transplant			
Variable	Auto-HSCT	10/10 UD-HSCT	9/10 UD-HSCT	*p*
Number (total: 2879)	1202	1302	375	
Gender, *n* (%)				0.046
Male	681 (57)	694 (53)	188 (50)	
Female	518 (43)	608 (47)	187 (50)	
WBC at diagnosis (×10^9^/l), median (range)	13.8 (0.3–820)	10 (0.3–900)	9.9 (0.2–790)	0.32
Missing	592	308	99	
Cytogenetic risk, *n* (%)				<10^−4^
Good	392 (33)	137 (11)	26 (7)	
Intermediate	624 (51)	550 (42)	165 (44)	
Poor	186 (16)	615 (47)	184 (49)	
Molecular aberrations, *n* (%)				
NPM1 mutation				0.001
Absent	64 (34)	150 (49)	41 (53)	
Present	124 (66)	154 (51)	37 (47)	
Missing	438	280	96	
FLT3-ITD				<10^−4^
Absent	159 (70)	178 (48)	48 (44)	
Present	68 (30)	197 (52)	61 (56)	
Missing	399	209	66	
CEBPA mutation				0.07
Absent	40 (82)	109 (90)	33 (97)	
Present	9 (18)	12 (10)	1 (3)	
Missing	577	463	140	
No. of induction courses to reach CR1, *n* (%)				<10^−4^
1	617 (51)	722 (56)	187 (50)	
More than 1	195 (17)	408 (31)	122 (33)	
Missing	390 (32)	172 (13)	66 (17)	
MRD status at transplant				0.53
MRD negative	361 (79)	352 (73)	81 (76)	
MRD positive	99 (21)	132 (27)	26 (24)	
Missing	742	818	268	
Median age at transplant, years (range)	49 (18–78)	51 (18–76)	49 (18–69)	0.004
Median interval diagnosis transplant, days (range)	158 (75–813)	174 (66–997)	177 (83–766)	<10^−4^
Median interval CR1 transplant, days (range)	109 (21–365)	115 (18–447)	121 (21–348)	0.41
Missing	390	172	66	
Median year of transplant (range)	2008 (05–13)	2010 (05–13)	2010 (05–13)	<10^−4^
Stem cell source, *n* (%)				<10^−4^
BM	53 (4)	258 (20)	58 (16)	
PBSCs	1149 (96)	1044 (80)	317 (84)	
TBI-including conditioning, *n* (%)				<10^−4^
No	1112 (93)	936 (72)	262 (70)	
Yes	85 (7)	364 (28)	113 (30)	
Conditioning intensity, *n* (%)				
MAC	–	619 (48)	194 (52)	
RIC	–	677 (52)	180 (48)	
Median follow-up, months (range)	45 (1–128)	36 (1–119)	25 (1–113)	

Legend: *BM* bone marrow, *CEBPA* CCAAT/enhancer-binding protein alpha, *CR1* first complete remission, *FLT3-ITD* fms-like tyrosine kinase-internal tandem duplication, *MAC* myeloablative, *MRD* minimal residual disease, *NPM1* nucleophosmin, *PBSCs* peripheral blood stem cells, *RIC* reduced-intensity, *TBI* total-body irradiation, *WBC* white blood cells

Since patient and disease characteristics were unevenly distributed among the transplant categories (auto-HSCT, 10/10 UD-HSCT, and 9/10 UD-HSCT), we fit a propensity score model generating ATT-weighted means for the three groups. After weighting, group characteristics were similar in terms of all baseline covariates used for PS estimation (Table 2).

**Table 2:** ATT-weighted means for transplant groups

	Weighted means			*p*	
Variable	Auto-HSCT	10/10 UD-HSCT	9/10 UD-HSCT	10/10 UD-HSCT vs auto-HSCT	9/10 UD-HSCT vs auto-HSCT
Global population					
Median age at transplant, years	47	46	47	0.84	1.00
Median year of transplant	2008	2008	2008	0.66	0.88
Median interval diagnosis transplant (days)	178	179	179	0.80	0.49
Good-risk cytogenetics (%)	33	31	30	1.00	1.00
Poor-risk cytogenetics (%)	15	17	19	1.00	1.00
More than 1 induction to achieve CR1 (%)	16	18	17	0.7	0.91
By cytogenetic risk					
Good risk					
Median age at transplant, years	44	44	n.a.^a^	0.96	n.a.
Median year of transplant	2009	2009	n.a.	0.56	n.a.
Median interval diagnosis transplant (days)	186	188	n.a.	1.00	n.a.
More than 1 induction to achieve CR1 (%)	0.01	0.09	n.a.	0.84	n.a.
Intermediate risk					
Patient age (years)	48	48	49	0.96	0.39
Year of transplant	2008	2008	2008	0.36	0.83
Interval diagnosis transplant (days)	174	181	183	0.51	0.90
More than 1 induction to achieve CR1 (%)	19	22	17	0.36	0.91
Intermediate-risk *wt*FLT3					
Patient age (years)	46	48	n.a.	0.75	n.a.
Year of transplant	2008	2009	n.a.	0.46	n.a.
Interval diagnosis transplant (days)	118	115	n.a.	0.93	n.a.
More than 1 induction to achieve CR1 (%)	17	21	n.a.	0.81	n.a.
Poor risk					
Patient age (years)	50	50	50	1.00	0.93
Year of transplant	2008	2008	2009	0.87	0.11
Interval diagnosis transplant (days)	172	170	173	1.00	0.91
More than 1 induction to achieve CR1 (%)	24	25	27	0.81	0.77

Legend: *ATT* average treatment effect among the treated, *CR1* first complete remission, *wtFLT3* wild-type FLT3

^a^In good risk and intermediate *wt*FLT3 categories, only auto-HSCT and 10/10 UD-HSCT were analyzed, as the number of 9/10 UD-HSCT transplants resulted too limited

### Outcome in the overall population

**Fig. 1:**
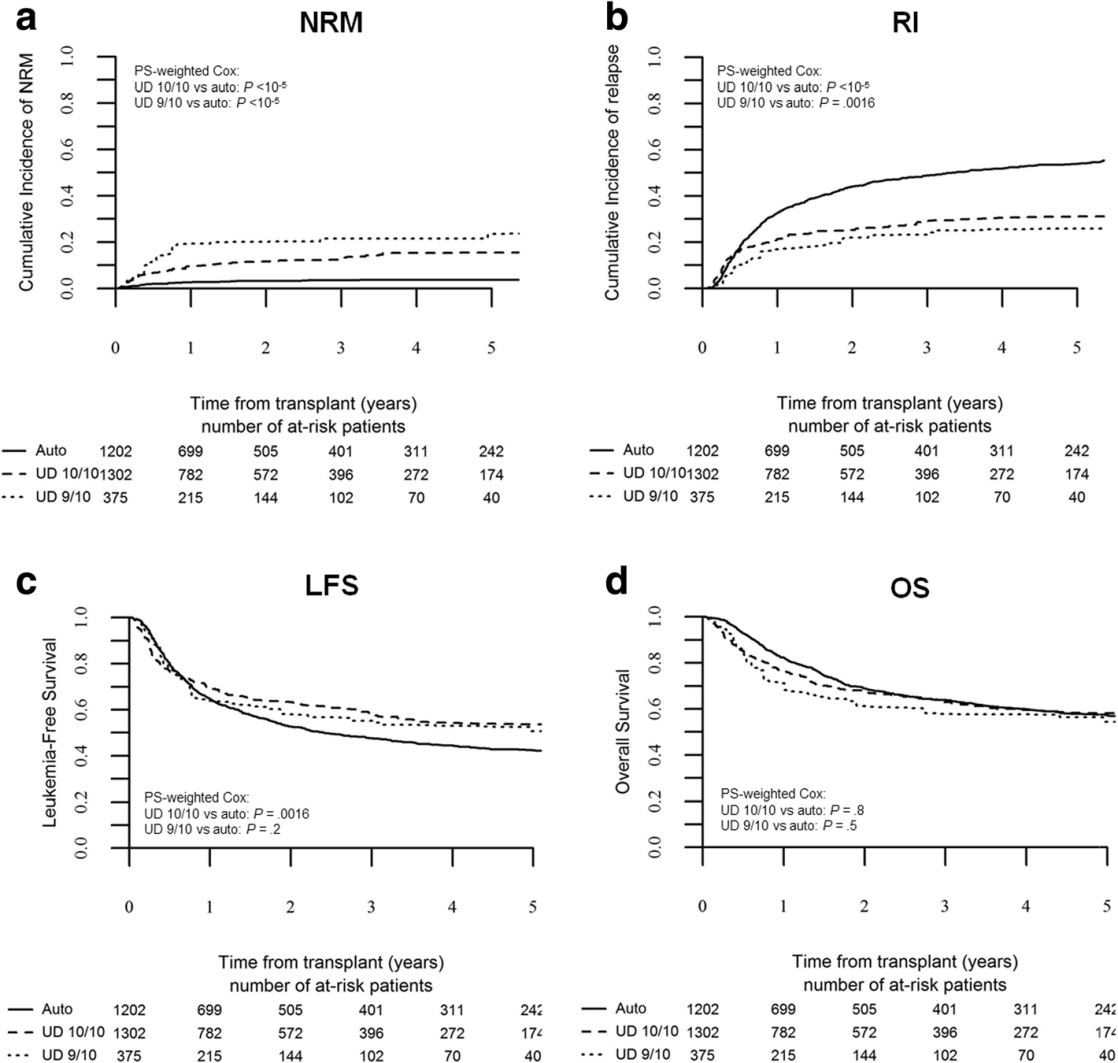
Outcome by type of transplant in the global population. The cumulative incidence of non-relapse mortality (**a**) and relapse (**b**) by transplant type; the probability of leukemia-free survival (**c**) and overall survival (**d**) in the global population. Kaplan-Meier curves and Cox analysis are weighted for propensity score; Cox analysis is further adjusted for kind of conditioning and stem cell source

In the global population, the 3-year NRM rate was significantly lower for auto-HSCT compared to 10/10 UD-HSCT and 9/10 UD-HSCT (being 4 ± 2, 13 ± 2, and 21 ± 3 %, respectively; Fig. 1a), as evidenced by PS-weighted Cox analysis (10/10 UD-HSCT vs auto-HSCT: HR 3.1, *p* < 10^−5^, 95 % CI 2–4.7; 9/10 UD-HSCT vs auto-HSCT: HR 4.5, *p* < 10^−5^, 95 % CI 2.5–8.1, Table 3). The 3-year RI was higher following auto-HSCT (49 ± 3 %) compared to 10/10 UD-HSCT (29 ± 3 %) and 9/10 UD-HSCT (23 ± 3 %), as evidenced by PS-weighted Cox analysis (10/10 UD-HSCT vs auto-HSCT: HR 0.5, *p* < 10^−5^, 95 % CI 0.4–0.7; 9/10 UD-HSCT vs auto-HSCT: HR 0.5, *p* = 0.0016, 95 % CI 0.3–0.8; Fig. 1b).

**Table 3:** PS-weighted Cox analysis of transplant outcomes, adjusted for kind of conditioning and stem cell source

Type of transplant	NRM			RI			LFS			OS		
	HR	95 % CI	*p*	HR	95 % CI	*p*	HR	95 % CI	*p*	HR	95 % CI	*p*
Global population												
Auto-HSCT (reference)	1			1			1			1		
10/10 UD-HSCT	3.1	2–4.7	<10^−5^	0.5	0.4–0.7	<10^−5^	0.7	0.6–0.9	0.0016	0.97	0.8–1.2	0.84
9/10 UD-HSCT	4.5	2.5–8.1	<10^−5^	0.5	0.3–0.8	0.0016	0.8	0.6–1.1	0.227	1.1	0.8–1.7	0.49
By cytogenetic risk												
Good risk												
Auto-HSCT (reference)	1			1			1			1		
10/10 UD-HSCT	1.9	0.7–5.5	0.24	0.5	0.3–0.9	0.018	0.7	0.4–1.1	0.1	1.1	0.6–2	0.7
Intermediate risk												
Auto-HSCT (reference)	1			1								
10/10 UD-HSCT	3.6	2–6.4	<10^−4^	0.5	0.4–0.7	<10^−5^	0.7	0.6–0.9	0.01	0.98	0.7–1.3	0.9
9/10 UD-HSCT	9.4	4.9–18	<10^−5^	0.4	0.3–0.8	0.004	1.1	0.7–1.6	0.7	1.6	1.001–2.5	0.049
Intermediate *wt*FLT3												
Auto-HSCT (reference)	1			1			1			1		
10/10 UD-HSCT	2.8	0.8–9.8	0.11	0.5	0.29–0.98	0.04	0.6	0.4–1.1	0.10	0.95	0.53–1.7	0.88
Poor risk												
Auto-HSCT (reference)	1			1			1			1		
10/10 UD-HSCT	6.3	2.3–17.4	0.0004	0.5	0.3–0.7	0.0003	0.7	2.3–17.4	0.0004	0.9	0.6–1.2	0.4
9/10 UD-HSCT	11.7	4–34.7	<10^−5^	0.7	0.4–1	0.08	1.03	0.7–1.5	0.88	1.3	0.9–1.9	0.2

Legend: *wtFLT3* wild-type FLT3

Fully matched UD-HSCT was associated with the best 3-year LFS (58 ± 3 %), while LFS rates were not statistically different between auto-HSCT and 9/10 UD-HSCT, being 48 ± 3 and 55 ± 3 %, respectively (10/10 vs auto-HSCT: HR 0.7, *p* = 0.0016, 95 % CI 0.6–0.9; 9/10 vs auto-HSCT: HR 0.8, *p* = 0.2, 95 % CI 0.5–1.1; Fig. 1c). The 3-year OS was not statistically different across the groups, being 64 ± 3, 63 ± 3, and 58 ± 4 % for auto-HSCT, 10/10 UD-HSCT, and 9/10 UD-HSCT, respectively (10/10 vs auto-HSCT: HR 0.98, *p* = 0.84, 95 % CI 0.8–1.2; 9/10 vs auto-HSCT: HR 1.1, *p* = 0.49, 95 % CI 0.8–1.7; Fig. 1d).

### Outcome by cytogenetic risk

**Fig. 2:**
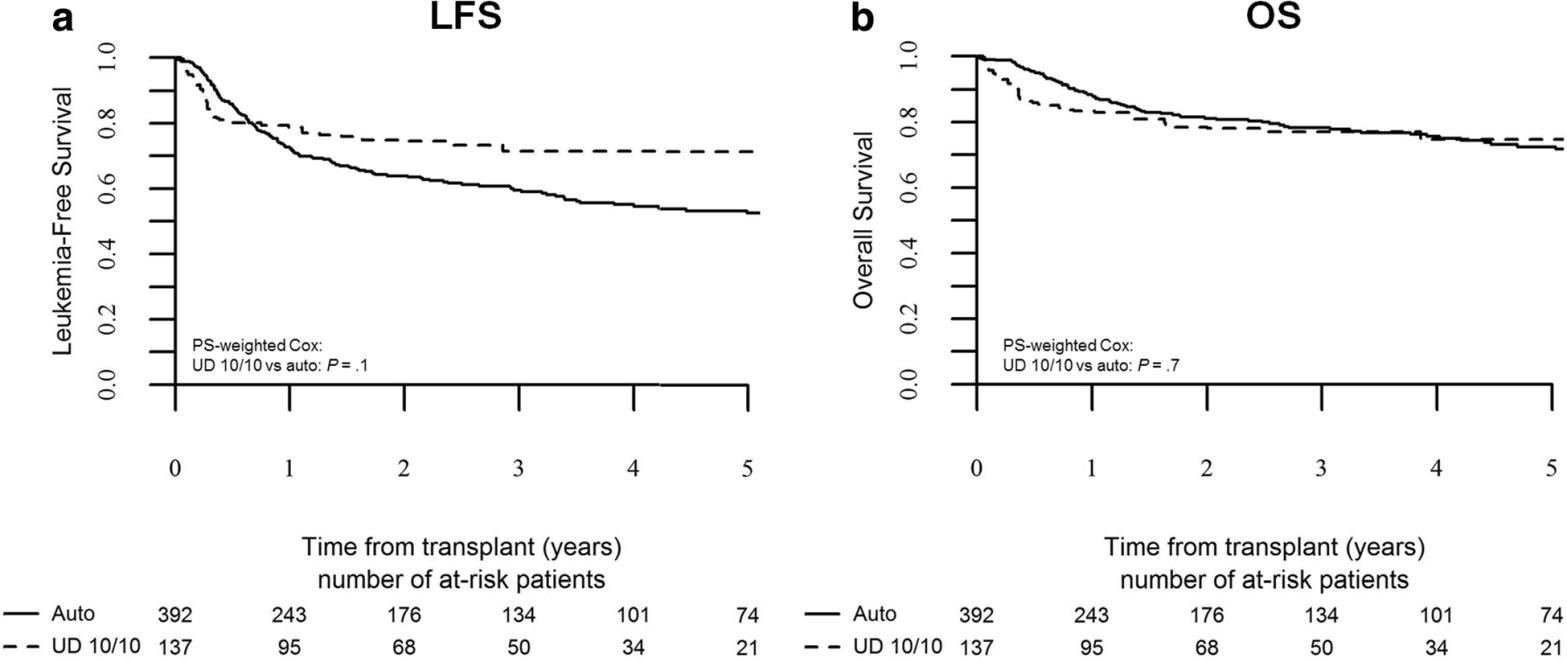
Leukemia-free survival and overall survival by type of transplant in good-risk patients. The probability of leukemia-free survival (**a**) and overall survival (**b**) in good-risk patients

In the favorable risk group, we could only compare outcome of auto-HSCT to 10/10 UD-HSCT, as the number of 9/10 UD-HSCT transplants was too limited. Auto-HSCT was associated with a 3-year RI rate of 36 ± 5 %, while 10/10 UD-HSCT provided a 3-year RI of 19 ± 5 %, which was significantly lower in PS-weighted Cox analysis (10/10 UD-HSCT vs auto-HSCT: HR 0.5, *p* = 0.018, 95 % CI 0.3–0.9). There was a trend for better 3-year LFS following 10/10 UD-HSCT compared to auto-HSCT, being 72 ± 6 and 59 ± 5 %, respectively (HR 0.7, *p* = 0.1, 95 % CI 0.4–1.1; Fig. 2a). Overall survival at 3 years was not significantly different, being 78 ± 4 % for auto-HSCT and 77 ± 5 % for 10/10 UD-HSCT (10/10 UD-HSCT vs auto-HSCT: HR 1.1, *p* = 0.7, 95 % CI 0.6–2; Fig. 2b).

**Fig. 3:**
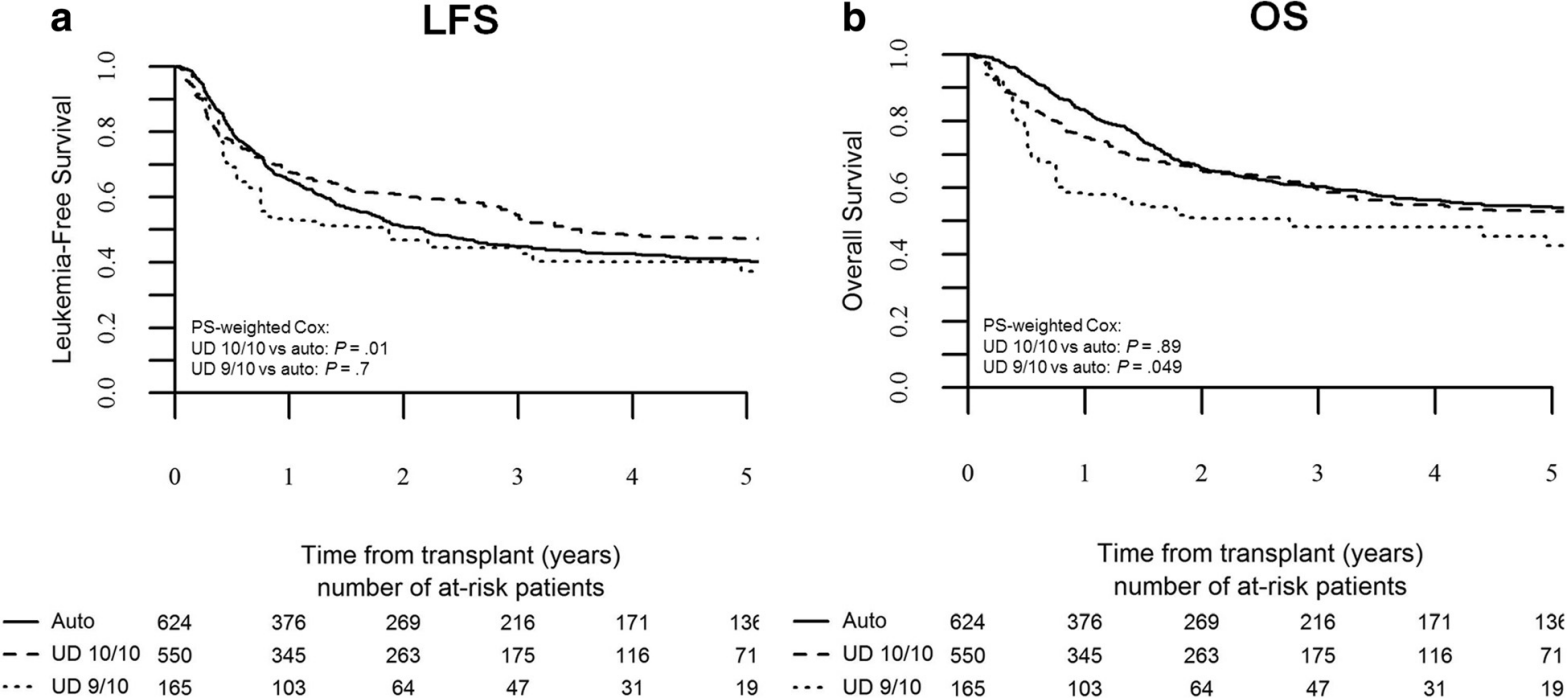
Leukemia-free survival and overall survival by type of transplant in intermediate-risk patients. The probability of leukemia-free survival (**a**) and overall survival (**b**) in intermediate-risk patients

Intermediate-risk AML represented the largest subpopulation in our survey and was the cohort in which the characteristics of the three groups showed the greatest overlap. In this subgroup, auto-HSCT was associated with higher relapse incidence (51 ± 4 %) compared to 10/10 UD-HSCT (30 ± 5 %) and 9/10 UD-HSCT (21 ± 4 %), as evidenced by PS-weighted Cox analysis (10/10 UD-HSCT vs auto-HSCT: HR 0.5, *p* < 10^−5^, 95 % CI 0.4–0.7; 9/10 UD-HSCT vs auto-HSCT: HR 0.4, *p* = 0.004, 95 % CI 0.3–0.8). NRM rates were significantly lower for auto-HSCT compared to 10/10 UD-HSCT and 9/10 UD-HSCT, being 4 ± 2, 16 ± 3, and 34 ± 5 %, respectively (10/10 UD-HSCT vs auto-HSCT: HR 3.6, *p* < 10^−4^, 95 % CI 2–6.4; 9/10 UD-HSCT vs auto-HSCT: HR 9.4, *p* < 10^−5^, 95 % CI 4.9–18). This translated to an advantage in terms of LFS for 10/10 UD-HSCT (54 ± 4 %) but not for 9/10 UD-HSCT (45 ± 5 %) over auto-HSCT (45 ± 4 %), as evidenced by PS-weighted Cox analysis (10/10 UD-HSCT vs auto-HSCT: HR 0.7, *p* = 0.01, 95 % CI 0.6–0.9; 9/10 UD-HSCT vs auto-HSCT: HR 1.1, *p* = 0.7, 95 % CI 0.7–1.6; Fig. 3a). Notably, 3-year OS did not differ between auto-HSCT (60 ± 4 %) and 10/10 UD-HSCT (60 ± 5 %), while it was significantly lower for 9/10 UD-HSCT (48 ± 4 %), as evidenced by PS-weighted COX analysis (10/10 UD-HSCT vs auto-HSCT: HR 0.98, *p* = 0.9, 95 % CI 0.7–1.3; 9/10 UD-HSCT vs auto-HSCT: HR 1.6, *p* = 0.049, 95 % CI 1.001–2.5; Fig. 3b).

Within the intermediate-risk cohort, we further analyzed the outcome of patients bearing wild-type FLT3; in this subpopulation, we could only compare auto-HSCT to 10/10 UD-HSCT, as the number of 9/10 UD-HSCT transplants was too limited to allow for propensity score weighting. RI rate was significantly higher for auto-HSCT compared to 10/10 UD-HSCT, being 55 ± 10 and 31 ± 12 %, respectively (10/10 UD-HSCT vs auto-HSCT: HR 0.5, *p* = 0.04, 95 % CI 0.3–0.9). Matched UD-HSCT was associated with a trend for better LFS compared to auto-HSCT, being 61 ± 11 and 41 ± 8 %, respectively (10/10 UD-HSCT vs auto-HSCT: HR 0.6, *p* = 0.10, 95 % CI 0.4–1.1), while no significant difference was observed in terms of OS (66 ± 10 and 60 ± 8 % for 10/10 UD-HSCT and auto-HSCT, respectively; 10/10 UD-HSCT vs auto-HSCT: HR 0.95, *p* = 0.88, 95 % CI 0.5–1.7).

**Fig. 4:**
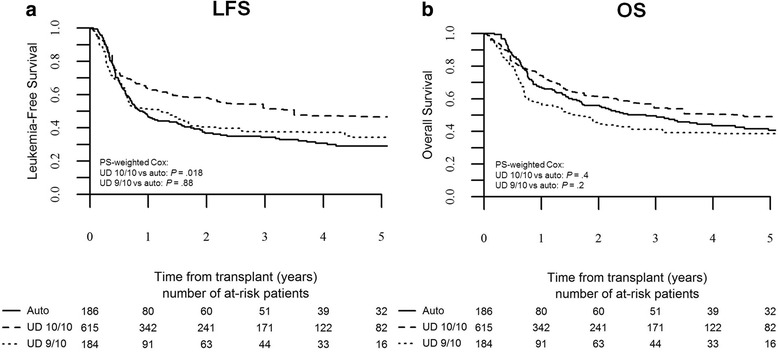
Leukemia-free survival and overall survival by type of transplant in poor-risk patients. The probability of leukemia-free survival (**a**) and overall survival (**b**) in poor-risk patients

In the poor-risk group, RI rate was once again significantly higher for auto-HSCT compared to 10/10 UD-HSCT and 9/10 UD-HSCT, being 64 ± 8, 34 ± 9, and 40 ± 9 %, respectively (10/10 UD-HSCT vs auto-HSCT: HR 0.5, *p* = 0.0003, 95 % CI 0.3–0.7; 9/10 UD-HSCT vs auto-HSCT: HR 0.7, *p* = 0.08, 95 % CI 0.4–1.1). Fully matched UD-HSCT was associated with better LFS compared to auto-HSCT, being 52 ± 8 and 34 ± 6 %, respectively (10/10 UD-HSCT vs auto-HSCT: HR 0.7, *p* = 0.018, 95 % CI 0.5–0.9), while LFS was not statistically different between auto-HSCT and 9/10 UD-HSCT, being 34 ± 6 and 38 ± 8 % (9/10 UD-HSCT vs auto-HSCT: HR 1, *p* = 0.88, 95 % CI 0.7–1.5; Fig. 4a). Overall survival was not statistically different across transplant groups, being 50 ± 7, 54 ± 8, and 41 ± 8 % for auto-HSCT, 10/10 UD-HSCT, and 9/10 UD-HSCT, respectively (10/10 UD-HSCT vs auto-HSCT: HR 0.9, *p* = 0.4, 95 % CI 0.6–1.2; 9/10 UD-HSCT vs auto-HSCT: HR 1.3, *p* = 0.2, 95 % CI 0.9–1.9; Fig. 4b).

### Outcome in the global registry population (6889 patients), unadjusted

As previously stated, from the starting 6889 patients receiving auto-HSCT, 10/10 UD-HSCT, or 9/10 UD-HSCT reported to the EBMT, 4010 patients were excluded due to incomplete cytogenetic data. We herein report the unadjusted results of outcome of all AML patients receiving auto-HSCT, 10/10 UD-HSCT, or 9/10 UD-HSCT in CR1 between 2005 and 2013 included in the EBMT registry: the 3-year LFS was 47 ± 2 % for auto-HSCT, 54 ± 2 % for 10/10 UD-HSCT, and 47 ± 4 % for 9/10 UD-HSCT, while the 3-year OS was 59 ± 2, 58 ± 2, and 50 ± 4 %, respectively.

### Outcome after the second transplant

Three hundred patients (25 % of the auto-HSCT group) received a subsequent allo-HSCT for leukemic relapse after auto-HSCT. Cytogenetic risk was good in 26 %, intermediate in 53 %, and poor in 21 % of the patients. With a median follow-up of 3.5 years after the second allograft, 2-year OS was 50 ± 6 %. OS was significantly affected by cytogenetic risk, being 61 ± 6 % in good risk, 45 ± 4 % in intermediate risk, and 49 ± 6 % in poor-risk patients (*p* = 0.019).

Conversely, 107 patients (7 % of the UD-HSCT group) underwent a second allo-HSCT for disease relapse post-first UD-HSCT transplant (79 in the 10/10 UD-HSCT group and 28 in the 9/10 UD-HSCT group). In this population, 2-year OS after second transplant was 25 ± 10 %.

### Acute and chronic graft-vs-host disease

Incidence of grade II–IV acute graft-vs-host disease (aGVHD) in patients receiving UD-HSCT was 27 ± 2 % in 10/10 UD-HSCT and 31 ± 4 % in 9/10 UD-HSCT, with no significant difference between the two groups (*p* = 0.1). Cumulative incidence of chronic graft-vs-host disease (cGVHD) at 2 years was 42 % in 10/10 UD-HSCT and 40 % in 9/10 UD-HSCT with no significant difference (*p* = 0.7). Incidence of severe (grade 3) cGVHD was also not different between the two cohorts, being 20 ± 2 and 17 ± 4 % in 10/10 UD-HSCT and 9/10 UD-HSCT, respectively (*p* = 0.16).

## Discussion

AML post-remission strategy remains largely debated. Different approaches are available, and recommendations are quickly mutating owing to continuous refining of risk stratification [13, 14], improvements in transplant preparatory regimens and GVHD prophylaxis [5, 15], and widening of the donor pool [16]. Therefore, when counseling a patient with AML in CR1, it is often difficult to make a straightforward statement.

Several randomized trials have shown significantly better LFS for auto-HSCT compared to chemotherapy as consolidation of remission in AML [17–20]. In the only prospective study conducted in the last decade, Vellenga et al. observed a reduced relapse rate following auto-HSCT when compared to chemotherapy [19]; the same group recently reported better survival following auto-HSCT in intermediate-risk AML [8]. Of note, in a recent report of a large randomized trial, Stone and colleagues [21] showed a significant survival benefit with the addition of the multi-target kinase inhibitor midostaurin to standard chemotherapy for AML patients bearing FLT3-ITD or TKD aberrations, an important finding that hopefully will pave its way into daily clinical practice.

Globally, donor vs no donor studies [22] and meta-analyses [23] evidenced a survival benefit for allo-HSCT over auto-HSCT in intermediate and poor cytogenetic-risk groups, but not in good-risk AML, in which the high NRM rate offsets the advantage of stronger anti-leukemic activity carried by allo-HSCT [24]. Nevertheless, donor vs no donor analyses suffered from biologic randomization bias; further, most studies combined patients receiving auto-HSCT and conventional chemotherapy in the no donor arm and included mostly young patients that received grafts from MSD, which accounts for a minority of transplants performed today. Furthermore, in some recent observations, auto-HSCT has been shown to provide similar survival to allo-HSCT from both sibling and unrelated donors [7–9]. Nonetheless, there is scarcity of literature confronting auto-HSCT to UD-HSCT, especially mismatched unrelated donor (MMUD).

We took therefore advantage of the EBMT-ALWP registry and analyzed a large homogeneous cohort of patients with AML in CR1. To mitigate the impact of the intrinsic limitations of a registry-based survey, such transplant-selection bias and disease risk imbalances between the groups, we performed a propensity score adjusted analysis, weighting transplant groups for the most significant patient characteristics, and further adjusting for kind of conditioning and stem cell source. Within this model, patients who received a UD-HSCT having significantly different characteristics compared to autografted patients had a very low impact on estimation of the outcome. In addition, we analyzed separately patients with good-, intermediate-, and poor-risk AML, to further elude the bias of cytogenetic risk unbalance*.* To better interpret the results obtained with PS-weighting analysis, it is worth noting that this model produces outcome results (i.e., LFS and OS) which, if compared to the crude (unadjusted) LFS and OS, are consistent for auto-HSCT, while better for UD-HSCT. This is a consequence of the rationale of the method itself which selects, among the UD-HSCT population, the patients which present similar characteristics to auto-HSCT patients.

Our data suggest that fully matched UD-HSCT provides better leukemia control but similar survival compared to auto-HSCT in AML in CR1. Further, mismatched UD-HSCT appears to be associated with inferior survival compared to auto-HSCT in patients bearing intermediate-risk cytogenetics.

The widespread availability of high-resolution HLA typing has greatly improved outcome of UD transplants, and results of allo-HSCT from fully HLA-matched UD are today overlapping with MSD outcome [25, 26]. However, MMUD transplants are associated with increased morbidity and mortality; in fact, higher incidence of both acute [27] and chronic [28, 29] GVHD rates have been described following mismatched transplants. In addition, NRM risk tends to increase proportionally to the number of HLA disparities [30–33], although improved outcome of MMUD transplants has recently been reported following RIC regimens [4]. Finally, recent developments in haploidentical transplantation are beginning to bring into question the choice of a mismatched unrelated over a haploidentical donor, when available [5].

Auto-HSCT results, on the other hand, have progressively improved. Switch of stem cell source from BM to peripheral blood stem cells (PBSCs) and refinements in preparatory regimens [15] have led to faster hematopoietic recovery, reduced mortality and satisfactory outcome; in a recent observation, Gorin et al. [34] reported a 2-year LFS of 61 % following auto-HSCT prepared with a busulfan-melphalan conditioning.

In a previous EBMT survey conducted on a cohort of patients affected by MDS or secondary AML, Al-Ali et al. [35] observed similar 3-year LFS and OS for 8/8 UD-HSCT and auto-HSCT; a landmark analysis revealed better outcome with MUD-HSCT only for patients surviving beyond 6 months since transplant. A more recent study by Cho et al. [36] analyzed a small population of young intermediate-risk AML patients undergoing either MSD, 8/8 UD-HSCT, or auto-HSCT; the authors reported an advantage in terms of LFS for 8/8 UD-HSCT over auto-HSCT, with no significant difference in OS. Similarly, in a very recent observation by Mizutani et al. [37], MUD-HSCT provided lower RI but no survival advantage over auto-HSCT in patients with intermediate-risk AML in CR1.

In the current study, we observed a significantly lower NRM and higher RI for auto-HSCT compared to UD-HSCT. In the global population, auto-HSCT provided an acceptable 3-year LFS rate of 48 %, which was significantly lower compared to 10/10 UD-HSCT, but not statistically different from 9/10 UD-HSCT. Nonetheless, the better leukemia control provided by fully matched UD-HSCT did not translate in a survival benefit, as OS at 3 years was similar for auto-HSCT and 10/10 UD-HSCT, while slightly lower for 9/10 UD-HSCT, this difference being not statistically significant.

In the subgroup analysis stratified by cytogenetic risk, auto-HSCT provided a particularly good outcome in patients with favorable risk AML, being 3-year LFS and OS rates 59 and 78 %, respectively; those results are consistent with previous reports [38]. There is evidence indicating that auto-HSCT is able to significantly reduce relapse risk in AML with favorable cytogenetics, which still carry disease recurrence rates up to 35–40 % following conventional chemotherapy, with a particular risk for core-binding factor (CBF) AML with adverse prognostic features [39] or positive MRD after consolidation chemotherapy [40]. Further, there is data suggesting that in NPM1-mutated and CEBPA double-mutated (CEBPAdm) AML, the high chemosensitivity of the disease might be exploited with auto-HSCT intensification [41, 42]. Awaiting for MRD-driven prospective trials comparing high-dose cytarabine and auto-HSCT in this setting, these findings support auto-HSCT as a valid strategy for consolidation of remission in patients with good-risk cytogenetics.

Intermediate risk represents the gray zone of AML guidelines. The role of allo-HSCT in these patients is not as clear as in poor-risk category [23], and it is becoming even more controversial with the incorporating of MRD data in clinical algorithms. In 2014, auto-HSCT was removed from NCCN recommendations in intermediate-risk AML, and today, most physicians would perform allo-HSCT in this setting, supported by a clear advantage in terms of LFS over auto-HSCT and conventional chemotherapy [23, 24]. However several studies, including recent analyses [7–9], failed to observe a survival advantage of allo-HSCT over auto-HSCT in intermediate-risk AML.

In our study, intermediate risk represented the largest subgroup, accounting for approximately half of all patients included in the analysis. Moreover, it was the cohort in which the characteristics of the three groups showed the greatest overlap and was therefore the main focus of our analysis. Forty-five percent of intermediate-risk patients who received auto-HSCT were alive and leukemia-free at 3 years after transplant. Further, in this subpopulation, matched UD-HSCT provided the best LFS, while no significant difference could be observed between auto-HSCT and 10/10 UD-HSCT in terms of OS. Similarly, in a subgroup analysis of patients bearing intermediate cytogenetics and *wt*FLT3, 10/10 UD-HSCT showed a trend for better LFS without a survival advantage over auto-HSCT. Notably, in the PS-weighted analysis conducted on the whole group of intermediate-risk patients, auto-HSCT provided better OS compared to 9/10 UD-HSCT. In a recent report by Cornelissen et al. [8], allo-HSCT was associated with better LFS compared to auto-HSCT, but similar OS was observed in intermediate-risk patients. In that study, only MSD or 8/8 UD-HSCT were allowed in intermediate-risk group; therefore, our observation of a survival advantage of auto-HSCT over MMUD in intermediate-risk AML can be interpreted as not in contradiction with previous data.

However, the good survival following auto-HSCT should be analyzed more carefully. Indeed while, as expected, OS rates of UD-HSCT were approximately 5 % higher than the respective LFS rates, in intermediate-risk patients receiving auto-HSCT, 3-year LFS was 45 %, while OS was as high as 60 %. This striking difference can be mostly explained as a consequence of successful salvage treatment for many patients relapsed after auto-HSCT. In fact, a considerably great proportion of patients who experienced disease recurrence following auto-HSCT were effectively rescued and received a subsequent allo-HSCT, which provided a 2-year OS of approximately 50 %.

Nevertheless, relapse incidence following auto-HSCT is disturbingly high and remains the biggest concern about this approach. We observed a 3-year cumulative RI of 51 % in intermediate-risk patients receiving an autograft. Most patients experienced disease recurrence within 2 years, but late relapses were noticed, as previously reported [15, 43]. The dynamic risk stratification allowed by MRD assessment is becoming crucial in AML post-remission setting [44, 45] and might help to identify the best candidates for auto-HSCT; in fact, auto-HSCT has been already proven able to provide long-term remission in MRD-negative APL [46] and ALL [47]. In the AML setting, this concept is currently under investigation in a prospective-MRD driven clinical trial by the *Gruppo Italiano Malattie EMatologiche dell’Adulto* (GIMEMA-AML1310) which results are awaiting.

Finally, quality of life of transplant survivors should be taken into account, since leukemia cure does not always coincide with full health. Different studies highlighted the high incidence of late effects after allo-HSCT, mostly but not only related to chronic GVHD [48]. In our survey, almost 40 % of UD transplant survivors experienced cGVHD, which was severe in approximately half of them. These data should be taken into consideration when comparing survival of auto-HSCT and UD-HSCT [49].

The current analysis has several limitations. First, as may occur in any multicenter registry study, the three transplant groups were unevenly balanced in terms of patient characteristics, and the retrospective design did not allow to study the reason for choosing UD-HSCT or auto-HSCT, which may vary according to physician and center strategy. We try to address those limitations fitting a PS-weighting model in order to control for the most significant pre-transplant covariates and further stratifying the analysis by cytogenetic risk. Additional limitations that are the consequence of being a registry-based study are missing data about molecular aberrations (i.e., NPM1, FLT3-ITD, and CEBPA status) and MRD status for part of the patients. However, it should be noted that NPM1 and FLT3 status was available in approximately one third of the patients with normal karyotype, which enabled us to perform an acceptable even if not optimal risk stratification.

## Conclusions

In conclusion, given the limitations of the study, in AML patients in CR1 lacking a MSD, 10/10 UD-HSCT significantly improves LFS, but this advantage does not translate in better OS compared to auto-HSCT. In intermediate-risk population, autologous transplant should be considered as a valid option, especially for patients lacking a fully HLA-matched donor, as better survival appears to be provided by auto-HSCT compared to mismatched UD transplant. Further, autologous transplant provided an encouraging outcome in favorable risk AML. These data may suggest that the current strategy for management of AML in CR1 should incorporate auto-HSCT in patients with good- and intermediate-risk cytogenetics, especially for those lacking a fully HLA-matched donor. Obviously, this strategy should be examined in well-designed multicenter randomized studies incorporating MRD status and center experience as well as novel approaches for post-transplantation maintenance as midostaurin or other novel compounds.

## Additional file

Additional file 1: Listing all EBMT members. (DOCX 17 kb)

## Abbreviations

ALWP: Acute leukemia working party
AML: Acute myeloid leukemia
ATT: Average treatment effect among the treated
auto-HSCT: Autologous stem cell transplantation
BM: Bone marrow
CBF: Core-binding factor
CEBPA: CCAAT/enhancer-binding protein alpha
CR1: First complete remission
EBMT: European society for blood and marrow transplantation
ELN: European leukemia net
FLT3-ITD: fms-like tyrosine kinase-internal tandem duplication
GIMEMA: Gruppo Italiano Malattie EMatologiche dell’Adulto
GVHD: Graft-vs-host disease
GVL: Graft-vs-leukemia
LFS: Leukemia-free survival
MAC: Myeloablative
MMUD: Mismatched unrelated donor
MRD: Minimal residual disease
MSD: Matched sibling donor allo-HSCT
MUD: Matched unrelated donor
NCCN: National Comprehensive Cancer Network
NPM1: Nucleophosmin
NRM: Non-relapse mortality
OS: Overall survival
PBSCs: Peripheral blood stem cells
PS: Propensity score
RI: Relapse incidence
RIC: Reduced-intensity
TBI: Total-body irradiation
WBC: White blood cells
wtFLT3: Wild-type FLT3
10/10 UD-HSCT: Unrelated donor transplantation matched at 10/10 HLA loci
9/10 UD-HSCT: Unrelated donor transplantation mismatched at a single HLA-locus
