# Supplementary material for: Matched and mismatched unrelated donor compared to autologous stem cell transplantation for acute myeloid leukemia in first complete remission: a retrospective, propensity score-weighted analysis from the ALWP of the EBMT
Source: J Hematol Oncol. 2016 Sep 2;9(1):79. doi: 10.1186/s13045-016-0314-x (PMC5009662; doi:10.1186/s13045-016-0314-x)
Supplement: Additional file 1: — Listing all EBMT members. (DOCX 17 kb) [file 13045_2016_314_MOESM1_ESM.docx]

**List of all EBMT members**

University Hospital, Essen, Germany; Helsinki University Central Hospital, Helsinki, Finland; Universitätsklinikum Dresden, Dresden, Germany; University of Freiburg, Freiburg, Germany; Hopital St. Louis, Paris, France; Deutsche Klinik für Diagnostik, Wiesbaden, Germany; University Hospital Eppendorf, Hamburg, Germany; Hannover Medical University, Hannover, Germany; University Hospital Leipzig, Leipzig, Germany; CHU Bordeaux, Pessac, France; Silesian Medical Academy, Katowice, Poland; Centre For Clinical Haematology, Birmingham, United Kingdom; Erasmus MC-Daniel den Hoed Cancer Centre, Rotterdam, Netherlands, The; Hopital E. Herriot, Lyon, France; Nottingham City Hospital, Nottingham, United Kingdom; University Medical Centre, Utrecht, Netherlands, The; Centre Pierre et Marie Curie, Alger, Algeria; University Hospital, Basel, Switzerland; Sezione di Ematologia, Perugia, Italy; University Hospital Gasthuisberg, Leuven, Belgium; Medizinische Klinik und Poliklinik, Ulm, Germany; Huddinge University Hospital, Huddinge, Sweden; Hopital Claude Huriez, Lille, France; Charles University Hospital, Pilsen, Czech Republic; Hopital de Purpan, Toulouse, France; Hopital Saint Antoine, Paris, France; University Medical Center St. Radboud, Nijmegen, Netherlands, The; Charité Universitätsmedizin Berlin, Berlin, Germany; Tel-Aviv University, Tel-Hashomer, Israel; King Faisal Specialist Hospital & Research Centre, Riyadh, Saudi Arabia; Leiden University Hospital, Leiden, Netherlands, The; Hôpital Henri Mondor, Creteil, France; CHU Nantes, Nantes, France; St. James Hospital Trinity College, Dublin, Ireland; University of Heidelberg, Heidelberg, Germany; Service d`Onco Hematologie, Strasbourg, France; Ospedali Riuniti di Bergamo, Bergamo, Italy; Klinikum Nürnberg, Nürnberg, Germany; Universität Tübingen, Tübingen, Germany; Hopital Jean Minjoz, Besancon, France; Hôpital Necker, Paris, France; Unité de transplantation et de thérapie cellulaire, Marseille, France; Royal Marsden Hospital, London, United Kingdom; GKT School of Medicine, London, United Kingdom; Klinikum Grosshadern, Munich, Germany; Policlinico San Matteo, Pavia, Italy; George Papanicolaou General Hospital, Thessaloniki, Thessaloniki, Greece; University of Münster, Münster, Germany; Erciyes Medical School, Kayseri, Turkey; Cliniques Universitaires St. Luc, Brussels, Belgium; Bologna University, S.Orsola-Malpighi Hospital, Bologna, Italy; Hopital A. Michallon, Grenoble, France; Gazi Universitesi Tip Fakültesi, Ankara, Turkey; University College London Hospital, London, United Kingdom; Univ.`La Sapienza`, Rome, Italy; CHU Lapeyronie, Montpellier, France; Hospital Pitie-Salpetriere, Paris, France; Ospedale di Careggi, Firenze, Italy; Hopital La Miletrie, Poitiers, France; University Hospital, Uppsala, Sweden; Ankara University Faculty of Medicine, Ankara, Turkey; University Regensburg, Regensburg, Germany; University Hospital, Lund, Sweden; Centre Henri Becquerel, Rouen, France; Hospital U. Marqués de Valdecilla, Santander, Spain; VU University Medical Center, Amsterdam, Netherlands, The; Bristol Royal Hospital for Children, Bristol, United Kingdom; Royal Victoria Infirmary, Newcastle-Upon-Tyne, United Kingdom; University of Liege, Liege, Belgium; University of Saarland, Homburg, Germany; Istituto Scientifico H.S. Raffaele, Milano, Italy; Department of Haematology, Oxford, United Kingdom; Philipps Universitaet Marburg, Marburg, Germany; UnitT de Transplantation MTdullaire, Vandoeuvre Les Nancy, France; Rome Transplant Network, Rome, Italy; Rikshospitalet, Oslo, Norway; Royal Free Hospital and School of Medicine, London, United Kingdom; Glasgow Royal Infirmary, Glasgow, United Kingdom; Ospedale San Martino, Genova, Italy; Klinik für Knochenmarktransplantation, Idar-Oberstein, Germany; Hopitaux Universitaires de Geneve, Geneva, Switzerland; Johannes-Gutenberg-University, Mainz, Germany; Imperial College, London, United Kingdom; CHRU, Service des Maladies du Sang, Angers, France; Medizinische Universität Wien, Vienna, Austria; Haematology Department, Leeds, United Kingdom; Sahlgrenska University Hospital, Goeteborg, Sweden; Addenbrookes Hospital, Cambridge, United Kingdom; Southampton General Hospital, Southampton, United Kingdom; Bone Marrow Transplant Unit L 4043, Copenhagen, Denmark; Hospital Santa Creu i Sant Pau, Barcelona, Spain; University Hospital, Linköping, Sweden; Christie NHS Trust Hospital, Manchester, United Kingdom; Hospital Clínico, Salamanca, Spain; Dept. Haematology and Stem Cell Transplant, Budapest, Hungary; Institut Jules Bordet, Brussels, Belgium; Institute of Hematology and Blood Transfusion, Prague, Czech Republic; Centre Hospitalier Universitaire de Rennes, Rennes, France; Hadassah University Hospital, Jerusalem, Israel; Charité - Campus Benjamin Franklin, Berlin, Germany; Fédération de Greffe de Moelle et de, Clermont-Ferrand, France; Div. Stem Cell Transplantation and Immunotherapy, Kiel, Germany; Royal Liverpool University Hospital, Liverpool, United Kingdom; Ospedale San Gerardo, Monza, Italy; ICO – Hospital Duran i Reynals, Barcelona, Spain; North Trent BMT Programme (Adults), Sheffield, United Kingdom; Hôpital de l`ARCHET I, Nice, France; Vilnius University Hospital `Santariskiu Klinikos`, Vilnius, Lithuania; Manchester Royal Infirmary, Manchester, United Kingdom; S. Bortolo Hospital, Vicenza, Italy; University Hospital Maastricht, Maastricht, Netherlands, The; Leicester Royal Infirmary, Leicester, United Kingdom; Medical University of Gdansk, Gdansk, Poland; University Hospital, Zürich, Switzerland; Azienda Ospedaliera S. Giovanni, Torino, Italy; Umea University Hospital, Umeå, Sweden; Ospedale di Niguarda Ca` Granda, Milano, Italy; University Hospital, Udine, Italy; Medical University Graz, Graz, Austria; Hôpital Percy, Clamart, France; St. Bartholomew`s and The Royal London Hospital, London, United Kingdom; Turku University, Turku, Finland; Ospedale V. Cervello, Palermo, Italy; A.Z. Sint-Jan, Brugge, Belgium; Universita degli Studi di Bari, Bari, Italy; Ospedale La Maddalena - Dpt. Oncologico, Palermo, Italy; Ospedale Ferrarotto, Catania, Italy; University Medical Center Groningen (UMCG), Groningen, Netherlands, The; Tel Aviv Sourasky Medical Center, Tel Aviv, Israel; Ospedale Civile, Pescara, Italy; Ospedale Maggiore di Milano, Milano, Italy; Birmingham Heartlands Hospital, Birmingham, United Kingdom; RP Group Royal Perth Hospital, Perth, Australia; University of Napoli, Napoli, Italy; Universitätsklinikum Göttingen, Gottingen, Germany; Hospital Universitari Germans Trias i Pujol, Barcelona, Spain; University Hospital Erlangen, Erlangen, Germany; Hopital Nord, Saint Etienne Cedex 2, France; Elisabethinen-Hospital, Linz, Austria; University of Wales, Cardiff, United Kingdom; Institut Gustave Roussy, Villejuif, France; Hospital Clinic, Barcelona, Spain; University of Cologne, Cologne, Germany; Hacettepe University, Ankara, Turkey; University Hospital, Olomouc, Czech Republic; Hospital Universitario La Fe, Valencia, Spain; University Hospital Brno, Brno, Czech Republic; Evangelismos Hospital, Athens, Greece; Constantiaberg Medi-Clinic, Cape Town, South Africa; Central Clinical Hospital, Warsaw, Poland; AK St. Georg., Hamburg, Germany; Heinrich Heine Universität, Düsseldorf, Germany; King Hussein Cancer Centre, Amman, Jordan; Centre Hospitalier Universitaire, Caen, France; Hospital Vall d`Hebron, Barcelona, Spain; Ege University Medical School, Bornova-Izmir, Turkey; University Med. Center, Ljubljana, Slovenia; CHU Morvan, Brest, France; Institute of Haematology and Blood Transfusion, Warsaw, Poland; Charles University Hospital, Hradec Králové, Czech Republic; Academisch Ziekenhuis bij de Universiteit, Amsterdam, Netherlands, The; Belfast City Hospital, Belfast, United Kingdom; Inst. Portugues de Oncologia do Porto, Porto, Portugal; Universita Cattolica S. Cuore, Rome, Italy; K. Marcinkowski University of Medical Science, Poznan, Poland; Rambam Medical Center, Haifa, Israel; Policlinico G.B. Rossi, Verona, Italy; SPb State I. Pavlov Medical University, St. Petersburg, Russia; H SS. Antonio e Biagio, Alessandria, Italy; Klinikum Augsburg, Augsburg, Germany; DCTK, Wroclaw, Poland; Canterbury Health Laboratories, Christchurch, New Zealand; Hosp. Reina Sofia, Córdoba, Spain; Friedrich-Schiller-Universität Jena, Jena, Germany; Azienda Ospedaliera, Reggio Calabria, Italy; Hospital Gregorio Marañón, Madrid, Spain; Patras University Medical School, Patras, Greece; Univ. Est. de Campinas/TMO/UNICAMP, Campinas, Brazil; University Hospital VUB, Brussels, Belgium; Hospital Clínico Universitario, Valencia, Spain; Antwerp University Hospital (UZA), Antwerp Edegem, Belgium; Azienda Ospedali Riuniti di Ancona, Ancona-Torrette, Italy; University Hospital Gent, Gent, Belgium; Plymouth Hospitals NHS Trust, Plymouth, United Kingdom; Inst. Portugues Oncologia, Lisboa, Portugal; Hospital Covadonga, Oviedo, Spain; Robert-Bosch-Krankenhaus, Stuttgart, Germany; Ernst-Moritz-Arndt-Universität Greifswald, Greifswald, Germany; Klinikum Rechts der Isar, Munich, Germany; Hospital Morales Meseguer, Murcia, Spain; Hospital `Virgen del Rocio`, Sevilla, Spain; Western General Hospital, Edinburgh, United Kingdom; ZNA, Antwerp, Belgium; Beilinson Hospital, Petach-Tikva, Israel; Hospital Aranzazu, San Sebastian, Spain; Dipartimento di Oncologia, dei trapianti e delle, Pisa, Italy; Ospedale S. Camillo-Forlanini, Rome, Italy; Hospital San Maurizio, Bolzano, Italy; Allogeneic Stem Cell Transplant Center, Würzburg, Germany; Baskent University Hospital, Adana, Turkey; University Hospital, Bratislava, Slovakia; Tartu University Hospital, Tartu, Estonia; St. George`s Hospital, London, United Kingdom; Guy`s Hospital, London, United Kingdom; Karadeniz Technical University, Trabzon, Turkey; Policlinico Le Scotte, Siena, Italy; Hospital de Gran Canaria `Dr Negrin`, Las Palmas De Gran Canaria, Spain; AZ. Spedali Civili- Brescia Universitu of Brescia, Brescia, Italy; Hospital de la Princesa, Madrid, Spain; Specialized Children`s Oncohematpology Hospital, Sofia, Bulgaria; Hospital del SAS, Cádiz, Spain; Haukeland University Hospital, Bergen, Norway; University Hospital Innsbruck, Innsbruck, Austria; Hospedale Nord, Taranto, Italy; Ankara Numune Education and, Ankara, Turkey; Medical University of Lublin, Lublin, Poland; Ospedale A. Businco, Cagliari, Italy; IRCCS, Casa Sollievo della Sofferenza, San Giovanni Rotondo, Italy; Az. Ospedaliera S. Croce e Carle, Cuneo, Italy; Clinica Puerta de Hierro, Madrid, Spain; Kliniken Essen Süd, Essen, Germany; NHS Grampian, Aberdeen, United Kingdom; Fundeni Clinical Institute, Bucharest, Romania; Uni. Modena, Policlinico, Modena, Italy; KLINIKUM BREMEN - MITTE, Bremen, Germany; Mazzoni Hospital, Ascoli Piceno, Italy; Martin-Luther-Universität Halle-Wittenberg, Halle, Germany; Pesaro Hospital, Pesaro, Italy; Istanbul Tip Fakueltesi, Istanbul, Turkey; Wroclaw Medical University, Wroclaw, Poland; Medical Park Hospitals, Antalya, Turkey; Military Medical Academy, Belgrade, Serbia and Montenegro; University of Milano, Milano, Italy; Shariati Hospital, Teheran, Iran; A.O.R.N. `SAN.G MOSCATI`, Avellino, Italy; Unità Operativa Oncoematologia Pediatrica, Pisa, Italy; P.O. `R. Binaghi`, Cagliari, Italy; Military Medical Academy, Warsaw, Poland; University of Medicine and Pharmacy, Timisoara, Romania; GATA BMT Center, Ankara, Turkey; Universität Rostock, Rostock, Germany; Osmangazi University, Fac. of Medicine, Eskisehir, Turkey; Sectia Clinica de Hematolgoie si, Targu-Mures, Romania; The Trustee of London Clinic, London, United Kingdom; Istituto Clinico Humanitas, Milano, Italy; Ankara Oncology Research & Education Hospital, Ankara, Turkey; Hospital Universitario Son Espases, Palma De Mallorca, Spain; Cerrahpasa Medical School, Istanbul, Turkey; Hospital Univ. Virgen de las Nieves, Granada, Spain; National Haematology Centre, Riga, Latvia; Hospital Ampang, Ampang, Malaysia; Klinikum Oldenburg, Oldenburg, Germany; Campus Charité Mitte, Berlin, Germany; Univ. of Torino, Torino, Italy; American University of Beirut, Beirut, Lebanon; Ankara Bayindir Hospital, Haematology BMT, Ankara, Turkey; Hospital de Navarra, Pamplona, Spain; Wellington Hospital, Wellington, New Zealand; Belorussian Centre for Paediatric, Minsk, Belarus; Heilig Hartziekenhuis, Roeselare, Belgium; Hospital C. Panico, Tricase (Lecce), Italy; Arcispedale S. Maria Nuova, Reggio Emilia, Italy; Medical Academy, Wroclaw, Poland; CHRU Limoges, Limoges, France; Clinic Frankfurt (Oder) GmbH of Internal Medicine, Frankfurt (Oder), Germany; Hospital Universitario Virgen de la Arrixaca, Murcia, Spain; Ospedale Civile SS. Giovanni e Paolo, Venezia, Italy; Alfred Hospital, BMT Programme, Melbourne, Australia; Schneider Children`s Medical Center of Israel, Petach-Tikva, Israel; Institute G. Gaslini, Genova, Italy; Ospedale San Gerardo, Monza, Italy; Klinikum Karlsruhe gGmbH, Karlsruhe, Germany; IRCCS Policlinico San Matteo, Pavia, Italy; Hospital Clinico Universitario, Santiago De Compostela, Spain; Hospital de Santa Maria, Lisboa, Portugal; St.Savas Oncology Hospital, Athens, Greece; Univ. di Palermo, Palermo, Italy; Azienda Ospedaliera Universitaria San Martino, Genova, Italy; Klinikum der Johann-Wolfgang Goethe Universität, Frankfurt am Main, Germany; C.H.U. Timone Enfants, Marseille, France; Univ. of Parma, Parma, Italy; Clinica di Oncoematologia Pediatrica, Padova, Italy; University Hospital Center Rebro, Zagreb, Croatia; CHNDRF, Charleroi, Belgium; University Hospital Motol, Prague, Czech Republic; University of Cape Town Faculty of Health Sciences, Cape Town, South Africa; Istituto per l`Infanzia `Burlo Garofolo`, Trieste, Italy; University Hospital, Tübingen, Germany; Jagiellonian University, Krakow, Poland; University Children`s Hospital, Graz, Austria; Cardarelli Hospital, Napoli, Italy; Hospital Ramon y Cajal, Madrid, Spain; Clínica Universitaria de Navarra, Pamplona, Spain; IHOP, Lyon, France; Ospedale San Carlo, Potenza, Italy; Kreiskrankenhaus Hameln, Hameln, Germany; Hospital Guglielmo da Saliceto, Piacenza, Italy; Albert Aberts Stem Cell Transplant Unit, Pretoria, South Africa; Adnan Menderes University Med. Faculty, Aydin, Turkey; Spedali Civili - Brescia, Brescia, Italy; European Institute of Oncology, Milano, Italy; Hospital Juan Canalejo, La Coruña, Spain; ITMO-Instituto de Transplante de Medula Osea, La Plata, Argentina; St. Anna Kinderspital, Vienna, Austria; Chaim Sheba Medical Center, Tel-Hashomer, Israel; Hospital Carlos Haya, Málaga, Spain; Pediatric University Teaching Hospital, Bratislava, Slovakia; Dokuz Eylül Universitesi, Izmir, Turkey; Royal Hospital for Sick Children, Glasgow, United Kingdom; Musgrove Park Hospital (Somerset), Taunton, United Kingdom; The Children`s Hospital at Westmead, Sydney, Australia; Marmara University, Istanbul, Turkey; Niño Jesus Children`s Hospital, Madrid, Spain; University of Jena, Jena, Germany; University Hospital, Collegium Medicum UMK, Bydgoszcz, Poland; Royal Liverpool Children`s NHS Trust, Liverpool, United Kingdom; Our Lady`s Hospital for Sick Children, Dublin, Ireland; University of Bologna, Bologna, Italy; Wilhelminenspital, Vienna, Austria; Pédiatrie et Génétique Médicale, Rouen, France.
